# Learned Overeating: Applying Principles of Pavlovian Conditioning to Explain and Treat Overeating

**DOI:** 10.1007/s40429-018-0207-x

**Published:** 2018-04-21

**Authors:** Karolien van den Akker, Ghislaine Schyns, Anita Jansen

**Affiliations:** Faculty of Psychology and Neuroscience, Department of Clinical Psychological Science, Maastricht University, P.O. Box 616, 6200 MD Maastricht, The Netherlands

**Keywords:** Pavlovian conditioning, Food cue reactivity, Overeating, Obesity, Food cue exposure therapy

## Abstract

**Purpose of Review:**

This review provides an overview of recent findings relating to the role of Pavlovian conditioning in food cue reactivity, including its application to overeating and weight loss interventions.

**Recent Findings:**

Both in the laboratory and in real life, cue-elicited appetitive reactivity (e.g., eating desires) can be easily learned, but (long-term) extinction is more difficult. New findings suggest impaired appetitive learning in obesity, which might be causally related to overeating. The clinical analogue of extinction—cue exposure therapy—effectively reduces cue-elicited cravings and overeating. While its working mechanisms are still unclear, some studies suggest that reducing overeating expectancies is important.

**Summary:**

Pavlovian learning theory provides a still undervalued theoretical framework of how cravings and overeating can be learned and how they might be effectively tackled. Future studies should aim to elucidate inter-individual differences in Pavlovian conditioning, study ways to strengthen (long-term) extinction, and investigate the working mechanisms of cue exposure therapy.

## Introduction

Overweight and obesity prevalence has reached dramatic proportions. It is estimated that currently, around 2.1 billion individuals worldwide are overweight (body mass index [BMI] > 25 kg/m^2^), of which 600 million are obese (BMI > 30 kg/m^2^) [1, 2]. Given that obesity is associated with an increased risk for diabetes, cardiovascular disease, psychological problems and various mental illnesses [3–6], and societies face exploding healthcare costs due to obesity-associated morbidity [7, 8], halting, and reversing the obesity “pandemic” has become a priority in public health.

It has been proposed that the “obesogenic” environment is a main contributor to weight gain and obesity, for it promotes a sedentary lifestyle and provides an abundance of easy-to-get high-calorie foods (e.g., [9, 10, 11•]. Specifically, it is assumed that food-associated cues play a major role in overconsumption by evoking appetitive responses (such as food cravings), motivating one to (over)eat. Human Pavlovian learning in such “food cue reactivity” has however received little attention in both scientific studies and obesity interventions, even though experts have emphasized its importance in overeating and its contribution to the difficulty in achieving long-term weight loss [12–15]. This review provides an overview of recent findings on Pavlovian learning in food cue reactivity and food intake, including its application to weight loss interventions.

## Food Cue Reactivity and Pavlovian Conditioning

The obesogenic environment includes numerous food cues, such as the smell of freshly baked bread when walking past a bakery or the sight of delicious Italian ice cream when hiking though the park. Exposure to food cues activates a central appetitive state [16], which consists of diverse responses: psychological (craving, urge, or desire to eat), physiological preparatory (e.g., increased salivation and insulin release), and neurocognitive (e.g., brain activation patterns, allocation of attentional resources). The physiological preparatory responses (cephalic phase responses) aid in digestion, absorption, and metabolism [17, 18], and overall, stronger cue-elicited appetitive responding is thought to promote (over)eating [11•]. Indeed, increased levels of food cue reactivity (e.g., cue-elicited desires to eat) have been associated with overeating, unsuccessful dieting, a higher BMI, and eating psychopathology [11•, 19–24].

Although food cue reactivity has a strong genetic component [25], Pavlovian conditioning plays an important role as well. Every time palatable food is consumed, the food can be associated with cues in the internal or external environment, and these cues then promote cue reactivity upon their next encounter. In Pavlovian terms, food entering the digestive system is an unconditioned stimulus (US), and once a cue (conditioned stimulus; CS) has become associated with the US, it can stimulate appetitive responses, i.e., food cue reactivity (conditioned response: CR), which promotes food intake [13, 21, 26]. For example, consider Carl who repeatedly eats crisps in the evening when watching his favorite TV show: this context (CS; watching his favorite TV show in the evening) may become associated with eating crisps (US) and if this is the case, just the CS, that is, just the look of Carl’s favorite TV show, will elicit a desire for the crisps and promote its intake. Theoretically, any cue can become associated with palatable food intake, including the sight and smell of food, a hormonal or other internal physical state like hunger or satiety, specific rituals, cognitions, emotions, and so on [21, 27–29]. Several laboratory studies have examined this Pavlovian learning account of food cue reactivity by repeatedly pairing an initially neutral stimulus (CS+) with tasty foods (such as a piece of chocolate or a sip of milkshake), and comparing the appetitive responses to this stimulus with the appetitive responses to another stimulus (CS−) which is never followed by food intake. Stimuli that have been examined in these studies include trays [30, 31], boxes [32], geometric figures [33, 34•], objects [35], negative emotions [36], and (virtual) environments/contexts [37–39]. A consistent finding is that after conditioning, the CS+ (vs. CS−) evokes conditioned responding, as reflected by heightened psychological (e.g., explicit eating expectancies, eating desires, CS+ liking) (e.g., [30, 38, 39], psychophysiological (e.g., salivation) [34•, 35, 40], and behavioral responding (e.g., approach tendencies, place preferences, food intake/choice) (e.g., [30, 36, 37, 41]). Findings also suggest that appetitive conditioning usually occurs quickly; in most studies, only 3 to 6 CS–US pairings were sufficient, while one study even reports evidence for successful acquisition after only one CS–US pairing [35].

Though these findings seem in line with a role for Pavlovian conditioning in food cue reactivity, a relevant question is whether the findings are ecologically valid. The laboratory experiments are generally conducted under highly controlled conditions with very few distractors present and using arbitrary stimuli as CSs. In contrast, in daily life, CSs concern non-arbitrary naturalistic stimuli, and the CS–US associations will, for example, be more spread over time and embedded within a more distracting environment. In a recent study, we therefore examined whether appetitive conditioning also occurs under real-life circumstances, using times of day as conditioned stimuli [42•]. Participants were tested over a period of 15 days in their natural environment, using a smartphone application for administering a questionnaire (eating expectancies, eating desires) and for instructing them to consume chocolate provided by the experimenter. At one specific time of day (CS+), participants always received a signal to complete the questionnaire and the instruction to consume chocolate, whereas on another time of day (CS−), participants only completed the questionnaire. Results indicated considerably heightened eating desires and eating expectancies to the chocolate-associated time of day (vs. CS−) from the fifth day forward [42•]. Thus, consistent with the findings of laboratory studies, this study shows that cue-elicited eating desires can be learned with relative ease in the natural environment. Associative learning processes might therefore play a critical role in the experience of many daily eating desires.

Given the potentially important role of Pavlovian learning in the onset and maintenance of obesity, several recent studies have examined whether overweight individuals differ in appetitive Pavlovian learning from normal-weight individuals. Interestingly, the findings point towards *impairments* in acquiring appetitive responses (US expectancies, US desires, CS liking/preference) to food-associated stimuli in overweight and obese individuals [34•, 43, 44•]. In two studies, this was reflected by reduced differential responding (i.e., smaller differences in responding between the CS+ and CS−) [34•, 44•]. These findings are intriguing and raise the question if and how such impairments are causally linked to overeating. It might be that heightened CS− responding (which drove the deficit reported in [44•] reflects an impaired ability to inhibit appetitive responses to stimuli signaling non-reward in an appetitive context, which, in the natural environment, might plausibly increase reactivity and overeating to stimuli not associated with food intake [45, 46]. The findings might also indicate a tendency towards overgeneralization in overweight and obese individuals [34•, 44•]: a reduced ability to discriminate between predictive and non-predictive stimuli is a core feature of overgeneralization, and heightened responding to the CS− could result from overgeneralization from the CS+ to the CS− [47]. In the example of Carl, overgeneralization of appetitive responding might imply that while he originally learns to associate the eating of crisps to a particular context (e.g., sitting on the couch in the evening and watch his favorite TV show), he is also cue reactive to a wider variety of stimuli that are similar to the original CS+ but are never actually paired with eating (e.g., other people’s living rooms, or watching TV in general). Overgeneralization thus can easily lead to a large number of reactivity-evoking stimuli, promoting frequent cravings and overeating [34•]. Further, reduced learning about the predictive value of a CS promotes the formation of context–US associations [48]. This can lead to contextual, rather than strong cue-specific, reactivity (e.g., to Carl’s living room).

Another study suggesting altered appetitive learning in obese individuals found an opposite pattern: successful acquisition of a swallowing response to a CS+ vs. CS− in overweight but (unexpectedly) not in normal-weight individuals was found. One may argue that findings reporting reduced learning could have been plausibly caused by overweight/obese individuals using implicit or explicit strategies that can lead to reduced learning about CS–US contingencies, like being reluctant to report appetitive desires [49], having mindsets that suppress appetitive responding [50], or suffering from a greater cognitive load due to thoughts about eating, dieting, and weight [44•]. Thus, more research is needed to examine differences in the acquisition of appetitive responses in overweight/obese versus normal-weight individuals, taking into account these potential explanations, while preferably assessing sensitive physiological indices of appetitive learning in addition to self-report measures.

To summarize, food cue reactivity has been shown to be related to overeating and weight gain and can partly be learned through Pavlovian learning principles. Laboratory studies have shown that relatively few CS–US pairings are sufficient to form CS–US associations, and that—consequently—encounters of CSs enhance food cue reactivity. While this model has also been established in real-life circumstances, it remains of interest to study whether overweight individuals might be different from normal-weight individuals in their appetitive conditioning capacity. Initial evidence suggests that overweight individuals show impairments in the acquisition of appetitive responses, forming an interesting direction for future investigations.

## Extinction of Appetitive Responding

Given that food cue reactivity is partly learned, it may be reduced through extinction, which refers to the decline in (appetitive) responding with repeated CS–noUS pairings after acquisition [13]. Extinction of appetitive responses in real life can be achieved during a diet: when CSs, such as a chocolate-associated time of day, or watching one’s crisp-associated favorite TV show in the evening, are deliberately no longer followed by food intake (noUS), food cue reactivity (e.g., eating desires) decreases over time. Several laboratory studies have indeed shown that conditioned responses (such as eating expectancies, eating desires) reduce with non-reinforced CS presentations (e.g., [32, 51]). However, studies have also consistently suggested that a complete extinction of eating desires is difficult to achieve: towards the end of extinction, eating desires to the CS+ often remain higher than to the CS− (e.g., [30, 31, 51]—even when experimentally eliminating US expectancies through explicit instructions after acquisition [52]. This difficulty to completely extinguish eating desires might partly explain why it is so difficult to stick to one’s diet. Back to the example of Carl: watching his crisps-associated favorite TV show in the evening while no longer eating crisps will take some time for desires for crisps to reduce, and as long as the cued desires are still present, not eating the crisps will be difficult. If extinction of appetitive responses (such as eating desires) is slow, this could diminish one’s chances to diet successfully [53]. In addition, it has been argued that partial (in contrast to continuous) reinforcement of food cues might be frequently practiced by a significant portion of unsuccessful dieters who try to abstain from eating in response to certain food cues but regularly fail [32]. Partial reinforcement is well known to result in slowed extinction of appetitive responses (the partial reinforcement extinction effect)—possibly leading to a greater difficulty to extinguish appetitive responding and hence, more risk of lapses during one’s dieting attempts.

Why are cue-elicited desires seemingly so difficult to extinguish? It has been proposed that the difficulty to extinguish eating desires could indicate the presence of separate response systems: possibly, US expectancies stem from a system that serves to prepare an organism for the incoming US, whereas eating desires might stem from another (hedonic/evaluative) system that is based on the mere activation of the US representation in memory and that is less sensitive to extinction [30, 52, 54–56]. The implication is that to reduce acquired eating desires more effectively, it may be fruitful to investigate techniques that specifically target this hedonic/evaluative response system (see e.g., [54]).

One important characteristic of extinction is that it does not reflect mere “unlearning” of the CS–US relationship; it largely reflects *new* learning of a second—contextually controlled—inhibitory association (CS–noUS) that competes with the original CS–US association [57]. So, building upon the example of Carl: practicing extinction of not eating crisps while watching the TV show leads to a novel association: namely that the TV show is no longer a predictor of crisps intake (CS–noUS), while the original association between the TV show and crisps intake also remains intact (CS–US). Consistent with this account, a large amount of (mostly animal and some human) data show that after extinction procedures, seemingly extinguished appetitive responses can return under certain conditions—as demonstrated by phenomena such as rapid reacquisition (the rapid return of responding with new CS–US pairings after extinction, e.g., when eating crisps while watching the TV show once after a diet), reinstatement (the return of responding when the US is provided after extinction – e.g., eating crisps again after a diet), renewal (the return of responding after extinction with a change in context, e.g., extinction of evening cravings during a holiday and returning home), and spontaneous recovery (the spontaneous return of responding when time has elapsed after extinction, i.e., after a period of successful dieting) [13, 52, 56]. Extinction is dependent on the context for expression, and therefore, a change in context may promote a return of conditioned responses [57, 58]. Various types of (internal and external) stimuli can provide such contexts [59, 60•]. For example, one might learn that recent CS–US pairings are part of the acquisition “context,” and recent CS–alone presentations are part of the extinction context. When providing renewed CS–US pairings, this returns an individual to the acquisition context, resulting in a rapid return of responding [58]. For example, Carl previously repeatedly consumed crisps in the evening may have (largely) extinguished his or her evening-crisp-cravings by refraining from eating crisps for a while (CS–alone presentations, extinction). However, after months of strictly sticking to the crisps-less diet, Carl has some friends over and consumes crisps again in the crisp-associated context (reinforcing the original CS–US association). This return to this original acquisition context may elicit a renewed craving for crisps in the evenings over the next days and risks the lapse turning into a full-blown relapse. The ease of conditioned responses to re-emerge after extinction might explain the observation that although a considerable proportion of dieters are able to achieve initial weight loss, only few are able to also successfully *maintain* their weight loss—most dieters regain the lost weight (or even more) (e.g., [61, 62]).

Thus, insights into mechanisms of extinction can explain why diets are often unsuccessful: first, dieters likely experience persistent eating desires even after having successfully refrained from intake for a while due to not reaching (full) extinction (which may be exacerbated by partial reinforcement extinction effects). Such prolonged eating desires could be (too) hard to resist over a longer period of time—promoting lapses in the diet that, in their turn, again strengthen the CS–US association. Second, when dieters finally achieve (partial) extinction of food cue reactivity, responding can easily return, thereby promoting (re)lapse. Therefore, the long-term effectiveness of dieting efforts and treatments might be considerably improved by focusing on strengthening extinction learning and diminishing the magnitude of returns of appetitive responses.

## Cue Exposure Therapy: Effects, Working Mechanisms, and its Optimization

Cue exposure therapy (CET) is the direct clinical analogue of experimental extinction. In CET, individuals who suffer from overeating or binge eating are repeatedly and long-lasting exposed to palatable food cues (CSs), while refraining from intake (i.e., CS–noUS). During CET sessions, participants are thus exposed to intake-predicting cues (CSs) such as seeing, smelling, and tasting the food, while for example being in an overeating-associated context, and/or while experiencing a specific emotion, though food intake is not permitted (noUS) and food is thrown away at the end of the session. In line with a learning-based interpretation of food cue reactivity and overeating, the (small-scale) studies that have been conducted on CET suggest it leads to substantial reductions in cue-elicited food cravings and binge eating in bulimic patients [63–68]. In overweight and obese samples, CET has also found to significantly reduce cue-elicited cravings, binge eating, eating in the absence of hunger, and improved (maintenance of) weight loss [69–75]. Studies examining its long-term efficacy are however sparse with mixed findings [63, 70, 75], suggesting that exposure protocols may require optimization. A number of techniques derived from learning theory may be suitable for this (for recent overviews, see [14]). Here, we focus on techniques that have received (recent) empirical investigation.

### Foods to Be Included in Cue Exposure Sessions

A consistent finding in CET studies is that after one to seven exposure sessions to a certain high-calorie food, intake of that specific food during an ad libitum intake test is decreased, compared with ad libitum intake after a control lifestyle intervention [72, 73, 75]. Several prior studies have additionally examined whether exposure effects generalize to (often personalized) high-calorie food items not explicitly included during exposure sessions. These studies found that, generally, exposure effects do not transfer to non-exposed foods [72, 73, 75]. The implication for CET is straightforward: to achieve clinically relevant effects on overeating and weight loss, the therapist should include all relevant food cues in the exposure sessions, i.e., those that are identified by the subject as cues for overeating.

### Occasional Reinforced Extinction

It has been argued that occasionally reinforcing the CS–US relationship during exposure therapy may lead to a reduced risk of relapse of binge and overeating [76]. Occasional reinforcements might reduce the risk of relapse is by allowing a reinforced trial (CS–US pairing) to become associated with extinction (CS–alone presentations), leading to a slower rate of reacquisition with renewed CS–US pairings (e.g., when one experiences a lapse in one’s diet) [76]. In CET, one way to implement this technique is by encouraging individuals to occasionally consume small amounts of food during cue exposure exercises, in order to learn that eating a small amount of food is no longer a cue for overeating [13]. In support, animal and human appetitive conditioning [51, 76], as well as fear conditioning studies [77, 78], has reported evidence for occasional reinforcements during extinction to reduce relapse of responding (i.e., rapid reacquisition). In studies on CET, occasional reinforced extinction has not yet been studied in isolation, but some studies have incorporated this technique in exposure sessions by letting participants take small bites of high-calorie food previously associated with overeating [74•, 75]. These studies provided indirect support for the effectiveness of this technique by showing that specific overeating expectancies—specified for taking one bite of the food—significantly reduced after CET vs. control. Nevertheless, examining the pure benefits of adding occasional reinforcements to CET in (sub)clinical samples awaits investigation.

### Working Mechanisms of CET

Gaining insight into the possible working mechanisms of CET could greatly help to optimize cue exposure protocols. It has been proposed that the reduction in cue-elicited eating desires could be a primary driver underlying CET’s effectiveness, which is why most prior CET studies have focused on achieving a reduction (or “*habituation*”) of eating desires during exposure sessions. That is, exposure sessions are usually terminated when cue-elicited cravings (e.g., [79], arousal [71], or anxiety levels (e.g., [65])) have reached a pre-determined threshold (e.g., 10 on a 100-point scale). Likewise, a large amount of fear studies aimed to achieve habituation of fear within exposure sessions (within-session habituation or WSH) and between exposure sessions (between-session habituation or BSH). It has for long been argued that treatment success was dependent on successful WSH and BSH [80, 81]. However, research pointed out that indices of habituation are not consistently related to better treatment outcome in anxiety disorders [82]. Instead of focusing on habituation of fear, it has been proposed that exposure sessions should be designed in a way that maximizes the violation of CS–US expectancies, since this should effectively strengthen the inhibitory CS–noUS association [83]. Specifically, exposure sessions should aim at maximizing the expectancy of the individual’s catastrophic event to occur. For example, exposure sessions for panic disorder should carefully introduce CSs, such as high heart rate and sweating, which maximize the expectancy of the US to take place—the heart attack. The larger the mismatch between perceived expectancy of the heart attack to occur and the actual absence of such a heart attack (noUS), the stronger expectancies are violated and hence the better strengthening of the CS–noUS association. Some experimental studies have indeed provided evidence for the benefits of focusing exposure sessions on expectancy violation, rather than habituation: panic disorder patients who received two sessions of exposure therapy aimed at the violation of danger expectancies reported fewer panic attacks, less anxiety, and less avoidance of fearful situations after therapy, compared to patients who received two sessions of habituation-focused exposure ( [84]; see also [85]).

It is likely that violation of CS–US expectancies also play a significant role in CET. For example, a dieter may verbalize the expectancy “If I feel exhausted and chocolate is available (CS), then I will lose control and eat the entire chocolate box (US).” To maximize violation of overeating expectancies during CET, a patient is exposed to the box of chocolate when feeling exhausted, while exposure is continued until “losing control” is no longer expected (for example by asking how much time it will take to lose control and continuing exposure slightly beyond this point). To answer the question whether expectancy violation or habituation determines the effectiveness of CET, it was studied whether the reduction of CS–US expectancies (used as index of the violation of CS–US expectancies) or WSH and BSH of cue-elicited eating desires were associated with the intake of food after exposure [72, 73, 74•, 75]. Data of four experimental studies, similar in design, were aggregated to overcome power issues (see Table 1). It was found that greater expectancy violation, but not habituation, was modestly but significantly associated with reduced intake of exposed food after exposure. Thus, focusing on the reduction of eating desires during CET might not be necessary for a food cue exposure session to be effective. Instead, the reduction of CS–US expectancies might be an important treatment target, like in anxiety disorders.

**Table 1:** Correlations between z-scores of kcal intake of foods included (exposed) and not included (non-exposed) in CET and within-session habituation (WSH) and between-session habituation (BSH) of eating desires, and expectancy violation among participants of four studies [72, 73, 74•, 75]

	WSH of eating desires *n* = 140	BSH of eating desires *n* = 94	Expectancy violation *n* = 150
z intake exposed food^a^	− 0.05	− 0.08	− 0.18*
z intake non-exposed food^2^	0.02	− 0.06	− 0.12

In all studies [72, 73, 74•, 75], WSH was calculated subtracting the end eating desire from the session’s peak eating desire (measured on a 100 mm Visual Analogue Scale [VAS]), and, in case of multiple exposure sessions, the average was calculated. BSH was calculated for three studies (which included 2 or more exposure sessions) by subtracting peak desires in the last sessions from peak desires in the first session. Expectancy violation was calculated by subtracting post-intervention expectancies from pre-intervention expectancies (measured on 100 mm VAS); in studies where expectancies about eating specific foods were asked, scores were averaged. As expectancies were assessed using a 5-point scale in [73], scores were transformed (1 = 0; 2 = 25; 3 = 50; 4 = 75; 5 = 100)

*p < .05; sample sizes of each correlation vary due to measurement differences between studies

^a^To compare the intake during the taste test in all studies, z-scores were calculated. Some differences between studies are worth mentioning: g*eneral* exposed foods were included in the taste tests described in [72, 73], while foods in [74•, 75] were *personally selected*. In [75], one general exposed food item was additionally included during the taste test, but no general non-exposed food item was included as comparison and therefore not included in the present investigation. Pure kcals were converted into z-scores for the data of [72, 74•, 75], while the intake in [73] was converted into a percentage of daily required energy intake for children, and then converted into z-scores

However, to critically evaluate whether expectancy violation and/or habituation are *causally* related to treatment success, it is necessary to manipulate them. In a recent study, two exposure conditions were compared to a no-treatment control condition [74•]. Participants in the exposure conditions either received exposure explicitly aimed at the habituation of eating desires (exposure habituation condition) or exposure explicitly aimed at the violation of CS–US expectancies (exposure expectancy violation condition). In all conditions, food intake was measured, while expectancy violation and habituation was also measured in both exposure conditions. Results indicated that although expectancy violation was stronger in the exposure expectancy violation vs. exposure habituation condition during exposure sessions, the reduction from pre- to post-therapy was equally strong in both conditions. Moreover, consumption during an ad libitum intake test was similarly reduced across the two exposure conditions relative to the control condition. These results might question the importance of explicitly manipulating the violation of expectancies during exposure (see also [52]). They indicate that although greater reductions of CS–US expectancies are related to better outcome, it might not be essential to *explicitly* tackle these CS–US expectancies during cue exposure to reduce them successfully, since focusing on the reduction of eating desires led to similarly reduced expectancies and food intake after the intervention. Interestingly, similar findings have also been documented in the anxiety domain. For example, patients who completed a purely behavioral treatment for animal phobias profited as much from the treatment as patients who received a cognitive-behavioral treatment, in which dysfunctional cognitions were challenged by behavioral tests [86, 87]. Moreover, the (similar) change in maladaptive cognitions in both treatment conditions was found to mediate the improvement [87], which generally fits with the findings in CET: changing overeating expectancies is important, while explicitly challenging these expectancies does not seem to be crucial.

To recapitulate, studies indicate that CET is a promising intervention to target cue-elicited cravings, overeating, binge eating, and weight, although long-term effects are not yet studied. CET may be further optimized by incorporating insights from learning theory. The working mechanism of CET is still unknown, though initial evidence suggests that the violation of US expectancies is necessary. It is clear that more work in this area is needed.

## Conclusion

Pavlovian learning theory provides a theoretical framework of how cravings and overeating can be learned and how they might be effectively tackled. Experimental studies indeed show that initially neutral stimuli—both in the laboratory and in real life—can easily elicit conditioned appetitive responding after a few stimulus-food pairings. Extinction of some appetitive responses (e.g., desires) seems more difficult to achieve, and even after successful extinction, appetitive responding remains prone to relapse—perhaps explaining the general difficulty to achieve successful long-term weight loss. Cue exposure therapy, the clinical equivalent of extinction, seems effective in lowering cue-elicited cravings, overeating, and binge eating, but still little is known about the most effective exposure strategies and its working mechanisms. Reductions in overeating expectancies seem to be important. Future research may aim to elucidate inter-individual differences in Pavlovian learning (e.g., as a function of weight status) and their consequences for eating behavior, to study how successful long-term extinction of appetitive responding is best achieved, as well as to examine CET’s working mechanisms.
